# Pradefovir mesylate and tenofovir amibufenamide for chronic hepatitis B: a systematic review and pragmatic treatment algorithm

**DOI:** 10.3389/fphar.2026.1862886

**Published:** 2026-08-24

**Authors:** Shuqi Yang, Liting Zhan, Zimei Wan, Xueping Yu

**Affiliations:** 1 Department of Infectious Disease, Clinical Medical Research Center for Bacterial and Fungal Infectious Diseases of Fujian Province, Fujian Medical University Affiliated First Quanzhou Hospital, Quanzhou, Fujian, China; 2 Key Laboratory of Screening and Control of Infectious Diseases (Quanzhou Medical College), Fujian Provincial University, Quanzhou, Fujian, China

**Keywords:** chronic hepatitis B, pradefovir mesylate, tenofovir alafenamide, nucleotide prodrug, renal and bone safety, dyslipidemia, drug selection, Asia-Pacific

## Abstract

**Background:**

Pradefovir mesylate (PDV; approved in China in 2024) and tenofovir amibufenamide (TMF; approved in China in 2021) are novel hepatocyte-targeted nucleotide prodrugs recently approved in China. Their clinical positioning relative to the guideline-endorsed benchmark tenofovir alafenamide (TAF) for chronic hepatitis B management remains undefined, particularly regarding renal/bone safety and drug-drug interaction profiles.

**Objective:**

To synthesize available evidence and propose a pragmatic treatment selection framework.

**Methods:**

Narrative review with systematic literature search of Phase 2/3 randomized trials and observational real-world cohort studies (≥48 weeks follow-up, n ≥ 100) comparing PDV or TMF with TDF or TAF, with explicit evidence quality classification. Methods: Systematic review with qualitative synthesis of Phase 2/3 trials and observational cohorts comparing PDV or TMF with TDF or TAF. Risk of bias (RoB 2, NOS) and GRADE certainty were assessed.

**Results:**

TMF demonstrated noninferior antiviral efficacy versus TDF through Week 96 with superior renal and bone safety. TMF demonstrated noninferior antiviral efficacy versus TDF through Week 96, with improved renal and bone safety profiles compared with TDF. Retrospective real-world comparisons suggest comparable efficacy to TAF; however, prospective confirmation is needed. PDV demonstrated comparable HBV DNA suppression to TDF at Week 24, with sustained efficacy and favorable safety through Week 96 in Phase 3 trials PDV showed comparable HBV DNA suppression to TDF at Week 24, with Phase 3 data at Weeks 48 and 96 now available in Chinese CHB patients. PDV’s CYP3A4-dependent activation creates clinically significant drug-drug interaction potential, whereas TMF and TAF utilize non-CYP pathways with favorable interaction profiles. PDV’s CYP3A4-dependent activation raises the theoretical possibility of drug-drug interactions based on *in vitro* metabolic data, whereas TMF and TAF utilize non-CYP pathways with favorable interaction profiles.

**Conclusion:**

Based on currently available evidence, TMF represents a potentially viable TAF alternative for patients with renal/bone risk or anticipated long-term therapy, though definitive therapeutic equivalence requires prospective head-to-head randomized trials. PDV remains an emerging option requiring cautious implementation pending further characterization of its long-term durability, resistance barrier, and drug-drug interaction profile. Conclusion: TMF may represent an alternative to TAF for patients with renal/bone risk or anticipated long-term therapy, though definitive equivalence requires prospective head-to-head randomized trials. PDV offers comparable short-term efficacy to TDF, with longer-term data now available; continued follow-up will define its role among nucleotide prodrugs.

## 1 Introduction

High-genetic-barrier nucleos(t)ide analogues (NAs) remain the standard of care for chronic hepatitis B (CHB), with many patients requiring lifelong therapy. As durable viral suppression has become routine, treatment choices are increasingly guided by patient-specific safety considerations and comorbidity profiles, with particular emphasis on renal and bone vulnerability, cardiometabolic risk, and polypharmacy complexities (EASL, 2025; Terrault et al., 2018; Chinese Medical Association, 2022).

Two novel nucleotide prodrugs—pradefovir mesylate (PDV) and tenofovir amibufenamide (TMF)—are engineered to enhance hepatic delivery while reducing systemic exposure. This review consolidates Phase 2/3 evidence and pharmacokinetic insights to contextualize the clinical roles of PDV and TMF relative to the guideline-endorsed benchmark tenofovir alafenamide (TAF), with particular emphasis on Asia-Pacific clinical practice patterns and treatment decision-making in the contemporary era. This systematic review consolidates Phase 2/3 evidence and pharmacokinetic insights to contextualize the clinical roles of PDV and TMF relative to the guideline-endorsed benchmark tenofovir alafenamide (TAF), with particular emphasis on Asia-Pacific clinical practice patterns and treatment decision-making in the contemporary era.

PDV (approved in China in 2024) and TMF (approved in China in 2021) represent nascent additions to the HBV treatment armamentarium. TDF remains the most widely prescribed nucleotide analogue globally, endorsed by WHO and major guidelines as a first-line agent with the highest barrier to resistance, extensive long-term safety data, and universal low-cost availability. In resource-limited settings, it constitutes the backbone of HBV treatment programs. TAF is endorsed as a preferred first-line agent by major international guidelines including EASL (2025) and AASLD (2018) (EASL, 2025; Terrault et al., 2018), whereas PDV and TMF are currently approved primarily within China and have not yet been incorporated into Western or pan-Asian international guidelines. However, given that renal and bone safety concerns are universal in long-term CHB management, characterizing these hepatocyte-targeted prodrugs has relevance beyond the Asia-Pacific region. Although long-term comparative trials against TAF are still maturing, daily clinical practice cannot await completion of all Phase 3 trials. This review therefore systematic review therefore synthesizes current best evidence—acknowledging its provisional nature—into a pragmatic bedside framework to inform treatment selection during this evidence-to-practice transition. We explicitly recognize that the parameters presented herein will be refined as definitive comparative data become available.

## 2 Methods

### 2.1 Search strategy and selection criteria

We conducted a narrative review with systematic literature search synthesizing evidence on hepatocyte-targeted nucleotide prodrugs for CHB. While we employed a systematic search strategy and dual independent screening to ensure transparency and reproducibility, this review was not designed as a full systematic review adhering to PRISMA 2020 guidelines. The rationale is threefold. First, the clinical literature on pradefovir mesylate and tenofovir amibufenamide remains nascent and limited, yielding insufficient homogeneous data for quantitative synthesis or meta-analysis. Second, the anticipated included studies comprise heterogeneous designs (randomized Phase 2/3 trials, prospective cohorts, and retrospective real-world studies) that preclude uniform risk-of-bias assessment across disparate methodologies. Third, the primary objective was to synthesize available evidence into a pragmatic clinical framework for treatment selection rather than to generate pooled effect estimates. Consequently, we classify this work as a narrative review with systematic literature search, acknowledging its provisional nature during an evolving evidence landscape. Systematic searches of PubMed, Embase, Web of Science, Cochrane Library, CNKI, and VIP Database (January 2000 to December 2025) employed the search strategy: “(pradefovir OR tenofovir amibufenamide OR TMF OR PDV) AND (chronic hepatitis B OR CHB)”. Major international and regional clinical practice guidelines (EASL, 2025, AASLD 2018, and the Chinese 2022 Guidelines) were also screened to contextualize current treatment paradigms (EASL, 2025; Terrault et al., 2018; Chinese Medical Association, 2022). During manuscript preparation, additional targeted searches were performed to identify recently published pivotal studies, including a Phase 3 pradefovir trial published in 2026, which were not captured by the initial systematic search. Given the recent regulatory approvals of pradefovir mesylate (China, 2024) and tenofovir amibufenamide (China, 2021), peer-reviewed clinical literature remains limited. This paucity reflects the nascent stage of clinical evaluation rather than incomplete literature retrieval.

Inclusion criteria: (1) randomized Phase 2/3 trials with active comparator arms (TDF or TAF); (2) prospective cohort studies with follow-up duration ≥48 weeks and sample size ≥100; (3) observational real-world studies with adequate reporting of baseline characteristics and safety outcomes; (4) peer-reviewed original articles or systematic reviews in English or Chinese.

Exclusion criteria: Conference abstracts, preprints, case reports, studies with follow-up <24 weeks, and commentaries without primary data.

We conducted a systematic review following PRISMA 2020 guidelines. This systematic review was not registered prospectively because the study was initially designed as a narrative mini-review and was reclassified to a systematic review with qualitative synthesis during peer review. A formal protocol was not published *a priori*.

Given the limited number of head-to-head randomized trials simultaneously comparing all three agents and significant clinical heterogeneity across included study designs (randomized Phase 2/3 trials, prospective cohorts, and retrospective real-world studies), quantitative meta-analysis was not performed; instead, we employed qualitative synthesis. Inclusion criteria were stratified by study design and clinical context: (EASL, 2025): Randomized Phase 2/3 trials with active comparator arms (TDF or TAF). Phase 2 trials were eligible if follow-up was ≥24 weeks, acknowledging the nascent clinical data for novel prodrugs. Phase 3 trials were eligible if follow-up was ≥48 weeks (Terrault et al., 2018). Prospective cohort studies with follow-up duration ≥48 weeks and sample size ≥100 (Chinese Medical Association, 2022). Retrospective real-world studies with adequate reporting of baseline characteristics and safety outcomes. For head-to-head comparative cohorts directly comparing TMF versus TAF, follow-up ≥24 weeks was accepted given the absence of direct randomized evidence; for all other retrospective studies, follow-up ≥48 weeks was required (Reddy et al., 2008). Peer-reviewed original articles or systematic reviews in English or Chinese. Exclusion criteria: Conference abstracts, preprints, case reports, non-comparative observational studies with follow-up <24 weeks, and commentaries without primary data.

Systematic searches of PubMed, Embase, Web of Science, Cochrane Library, CNKI, and VIP Database (January 2000 to December 2025) employed the search strategy: “(pradefovir mesylate OR tenofovir amibufenamide OR TMF OR PDV OR tenofovir alafenamide OR TAF OR tenofovir disoproxil fumarate OR TDF) AND (chronic hepatitis B OR CHB OR hepatitis B virus OR HBV)”. Complete search strategies for each database are detailed in Supplementary Material Table S3. Major international and regional clinical practice guidelines (EASL, 2025, AASLD 2018, and the Chinese 2022 Guidelines) were also screened to contextualize current treatment paradigms (EASL, 2025; Terrault et al., 2018; Chinese Medical Association, 2022). During manuscript preparation, additional targeted searches were performed to identify recently published pivotal studies, including a Phase 3 pradefovir trial published in 2026, which were not captured by the initial systematic search. Duplicate records were identified and removed using EndNote 20 (Clarivate) automated deduplication, followed by manual verification of potential duplicates across Chinese and English databases. A PRISMA 2020 study selection flowchart is provided in Supplementary Material Table S1. Given the recent regulatory approvals of PDV (China, 2024) and TMF (China, 2021), peer-reviewed clinical literature remains limited. This paucity reflects the nascent stage of clinical evaluation rather than incomplete literature retrieval.

### 2.2 Study selection process

Two authors independently screened titles and abstracts, followed by full-text review of potentially eligible studies. Discrepancies were resolved by consensus or consultation with a third author. Evidence quality was characterized explicitly, with distinction between randomized controlled trials, prospective cohorts, and retrospective observational studies.

### 2.3 Data synthesis

Given the absence of direct head-to-head randomized trials simultaneously comparing PDV, TMF, and TAF, we employed narrative synthesis anchored to TDF as the common comparator. We explicitly classified evidence by type (RCT vs. observational) and follow-up duration. Safety outcomes were categorized by organ system (renal, bone, hepatic, metabolic), and clinical applicability was assessed within the context of Asia-Pacific treatment guidelines and polypharmacy patterns. We acknowledge that this framework is provisional and intended to guide clinical decision-making during an evolving evidence landscape.

Included studies comprised randomized trials (ranging from 24 to 96 weeks) and observational cohorts (24–48 weeks), reflecting stratified inclusion criteria by study design. Because no direct head-to-head randomized trial has simultaneously compared all three agents, we anchored cross-agent comparisons to TDF as the common comparator. We explicitly classified evidence by type (RCT vs. observational) and follow-up duration. Safety outcomes were categorized by organ system (renal, bone, hepatic, metabolic), and clinical applicability was assessed within the context of Asia-Pacific treatment guidelines and polypharmacy patterns. We acknowledge that this framework is provisional and intended to guide clinical decision-making during an evolving evidence landscape.

### 2.4 Risk of bias and certainty of evidence assessment

Randomized trials were evaluated using the Cochrane Risk of Bias 2.0 (RoB 2) tool across five domains. Observational real-world studies were assessed with the Newcastle-Ottawa Scale (NOS). The overall certainty of evidence for each major conclusion was graded using GRADE (high, moderate, low, very low), considering risk of bias, inconsistency, indirectness, imprecision, and publication bias. Detailed ratings are presented in Supplementary Material Table S2.

## 3 Results and discussion

### 3.1 Pharmacology and mechanistic distinctions among nucleotide prodrugs

PDV, TMF, and TAF represent distinct approaches to hepatocyte-targeted nucleotide delivery, each employing unique bioactivation pathways with important clinical consequences (EASL, 2025; Terrault et al., 2018; Chinese Medical Association, 2022).

PDV is a liver-targeted prodrug of adefovir developed using HepDirect technology to preferentially deliver the active moiety to hepatocytes while widening the therapeutic index relative to adefovir dipivoxil (Reddy et al., 2008). Following oral administration, PDV undergoes hepatic uptake and CYP3A4-mediated oxidative metabolism, releasing the active adefovir moiety preferentially within hepatocytes while minimizing systemic exposure. Quantitatively, the active adefovir moiety (PMEA) has a plasma terminal elimination half-life of 7.48 ± 1.65 h (Gilead Sciences, 2012), with an intracellular adefovir diphosphate (PMEA-DP) half-life of 12–36 h in lymphocytes (Tan et al., 2025); by comparison, plasma tenofovir following TAF administration exhibits a longer terminal half-life of approximately 32–40 h (Podany et al., 2018; Brooks et al., 2021). This obligate CYP3A4-dependent activation distinguishes PDV from other nucleotide prodrugs and creates potential for clinically significant pharmacokinetic interactions. This obligate CYP3A4-dependent activation distinguishes PDV from other nucleotide prodrugs and raises the theoretical possibility of pharmacokinetic interactions that warrant clinical verification. *In vitro* studies demonstrate potent inhibition of pradefovir-to-PMEA conversion by strong CYP3A4 inhibitors (Ki = 12.5 nM), while strong inducers may reduce hepatic bioavailability and therapeutic efficacy (Lin et al., 2006).

The competitive inhibition kinetics of CYP3A4 warrant additional mechanistic consideration. As a high capacity enzyme with broad substrate specificity, CYP3A4 concurrently metabolizes numerous coadministered drugs that function as competing substrates (Galetin et al., 2003). Fluctuating plasma concentrations of these medications, particularly agents with short half lives or extensive hepatic first pass metabolism, may transiently occupy CYP3A4 catalytic sites during peak absorption phases. This competitive binding could theoretically diminish diminishes the oxidative conversion of pradefovir to PMEA, potentially yielding resulting in suboptimal intracellular levels of PMEA-DP within hepatocytes. Sustained exposure to subtherapeutic intracellular active metabolite concentrations risks incomplete suppression of viral replication, generating pharmacological selection pressure that could theoretically promote the emergence of antiviral resistant HBV variants Sustained exposure to subtherapeutic intracellular active metabolite concentrations could, in theory, result in incomplete suppression of viral replication and generate pharmacological selection pressure that might promote the emergence of antiviral-resistant HBV variants (Zoulim and Locarnini, 2009). This mechanistic concern extends the interaction profile of pradefovir beyond formally classified strong CYP3A4 inhibitors to encompass any coadministered CYP3A4 substrate with sufficient competitive affinity, underscoring the necessity of comprehensive medication reconciliation prior to PDV initiation. However, this concern is extrapolated from *in vitro* CYP3A4 inhibition data and has not been validated in clinical trials. Given this theoretical possibility, medication reconciliation focusing on strong CYP3A4 inhibitors and inducers is prudent prior to PDV initiation, while acknowledging that clinical validation of these interactions in CHB populations remains pending.

Clinical validation of these drug-drug interaction risks in CHB cohorts is still pending. In healthy-volunteer studies and phase 2 randomized trials, PDV demonstrated acceptable safety and antiviral efficacy through Week 24 that was comparable to TDF In Phase 2 (Week 24) and Phase 3 (Weeks 48 and 96) randomized trials, PDV demonstrated antiviral efficacy comparable to TDF with acceptable safety (Gao et al., 2022) (Gao et al., 2026). While *in vitro* data suggest CYP3A4 interaction potential, clinical validation in CHB cohorts is pending. A recent randomized, double-blind, phase 3 noninferiority trial in Chinese patients with chronic hepatitis B comparing PDV versus TDF has now been published, providing extended efficacy and safety data beyond Week 24.

TMF represents a contrasting bioactivation strategy, being engineered to reduce systemic tenofovir exposure while maintaining antiviral potency. Unlike PDV, TMF bypasses CYP-mediated metabolism entirely, undergoing intracellular activation by non-CYP hepatic carboxylesterase 1 to generate tenofovir diphosphate with minimal systemic drug levels. This non-CYP activation pathway eliminates concerns regarding CYP3A4-mediated drug-drug interactions. Randomized phase 3 trials demonstrated noninferior antiviral efficacy versus TDF at Week 48, with improved renal and bone safety that persisted through Week 96 (Liu et al., 2021; Liu et al., 2023). Prospective real-world studies have reported largely neutral lipid profiles over 48 weeks (Chen et al., 2024; Peng et al., 2023).

Because TMF activation is absolutely dependent on hepatic CES1, the impact of advanced cirrhosis on this enzyme warrants mechanistic clarification. In cirrhotic livers, CES1 expression and activity are attenuated by both quantitative loss of functional hepatocytes and inflammation-driven transcriptional suppression. Experimental studies demonstrate that lipopolysaccharide-induced immunological liver injury significantly downregulates hepatic CES1 mRNA and protein expression, markedly impairing prodrug hydrolysis (Zhang et al., 2015). Mechanistically, inflammatory cytokines such as IL-33 trigger macrophage-derived exosomal miR-27b-3p, which targets Nrf2 and suppresses CES1 expression in hepatocytes, providing a molecular pathway for enzymatic downregulation beyond mere cell loss (Gao et al., 2023). Despite this attenuation, residual functional hepatocytes appear to retain sufficient CES1 catalytic capacity for effective TMF bioactivation, as evidenced by comparable 48-week virological response rates between TMF and TAF in recent prospective cohorts of decompensated cirrhosis (Rong et al., 2025).

TAF, the guideline-endorsed benchmark nucleotide analogue, is supported by robust phase 3 evidence demonstrating noninferior antiviral efficacy versus TDF and superior renal and bone outcomes through Week 96 supported by extensive phase 3 evidence demonstrating noninferior antiviral efficacy versus TDF and improved renal and bone safety profiles compared with TDF through Week 96 (Chan et al., 2016; Buti et al., 2016; Agarwal et al., 2018). TAF undergoes hydrolysis by lysosomal cathepsin A in hepatocytes to release tenofovir, achieving high intracellular concentrations with approximately 90% lower circulating tenofovir levels compared with TDF. Like TMF, TAF does not rely on CYP enzymes for bioactivation, conferring low drug-drug interaction potential in CHB monotherapy. Real-world cohorts report that TAF is associated with modest increases in total cholesterol and triglycerides compared with TDF, with variable effects on HDL and LDL (Lin et al., 2024).

**FIGURE 1:**
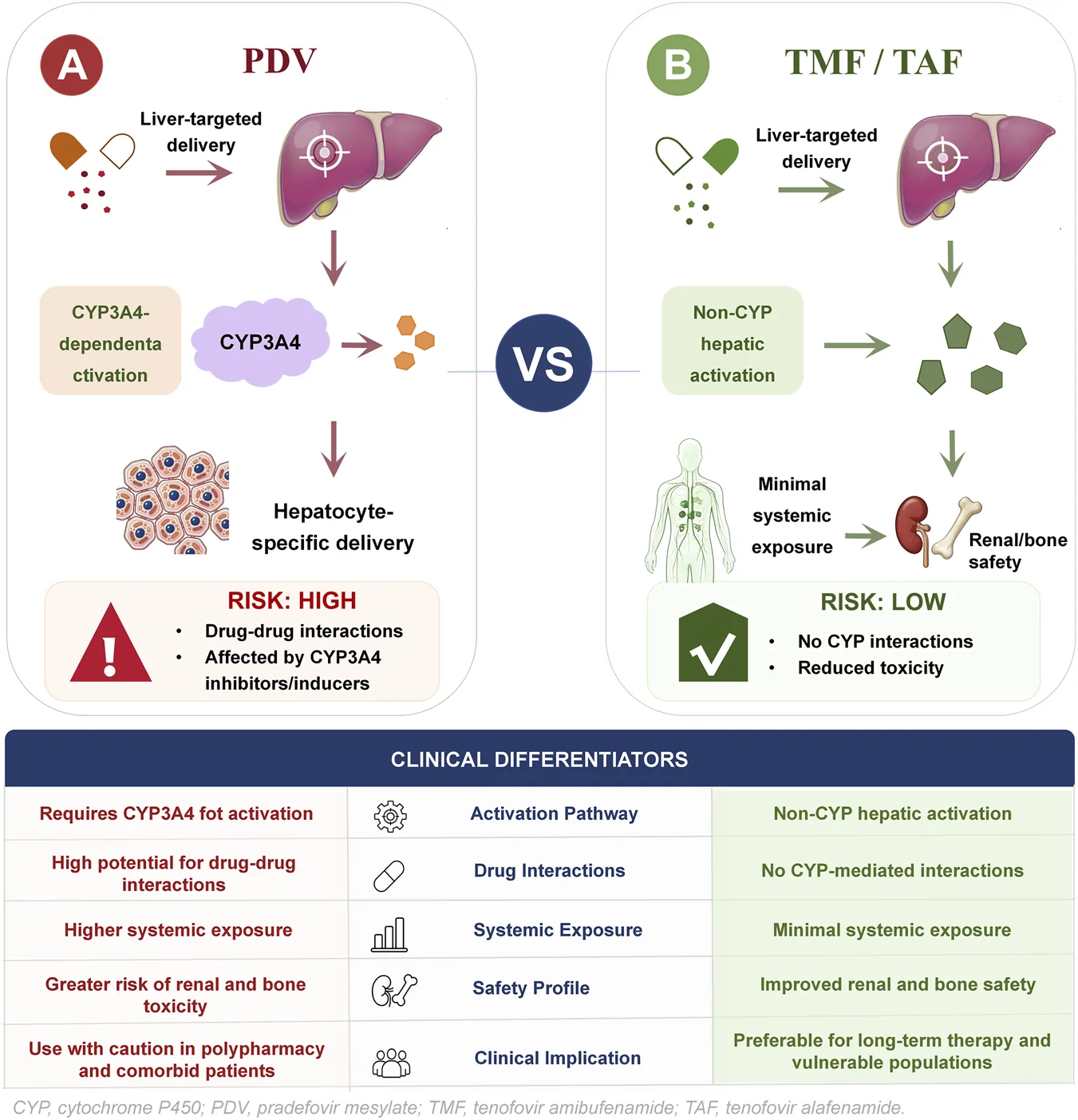
Mechanistic pathways and clinical differentiators of nucleotide prodrugs in chronic hepatitis **(A,B)** Pradefovir mesylate (PDV): A liver-targeted adefovir prodrug activated via obligate CYP3A4-mediated oxidation, resulting in hepatocyte-specific delivery of adefovir diphosphate (PMEA-DP). This CYP-dependent activation creates potential for clinically significant drug-drug interactions with strong CYP3A4 inhibitors (Ki = 12.5 nM) or inducers. Common CYP3A4 inhibitors include azole antifungals, macrolide antibiotics, and protease inhibitors; inducers include rifampicin and phenytoin. **(B)** Tenofovir amibufenamide (TMF) and tenofovir alafenamide (TAF): Both are tenofovir-based prodrugs activated via non-CYP hepatic pathways (carboxylesterase 1 for TMF; cathepsin A for TAF), generating tenofovir diphosphate (TFV-DP) with minimal systemic drug exposure. This non-CYP activation eliminates concerns regarding CYP-mediated drug interactions and confers renal and bone safety advantages through markedly reduced systemic tenofovir levels.

These mechanistic distinctions carry important clinical implications for polypharmacy scenarios. PDV’s absolute reliance on CYP3A4 necessitates careful prescreening for strong CYP3A4 inhibitors or inducers, whereas both TMF and TAF offer substantially greater pharmacologic flexibility in patients receiving CYP-modifying medications. Because PDV activation depends on CYP3A4, clinicians should consider screening for strong CYP3A4 inhibitors or inducers based on *in vitro* metabolic data; this precaution reflects a theoretical concern pending prospective clinical validation. Both TMF and TAF offer substantially greater pharmacologic flexibility in patients receiving CYP-modifying medications. Figure 1 summarizes these mechanistic pathways and highlights the divergent clinical consequences of CYP-dependent versus non-CYP activation strategies.

### 3.2 Clinical evidence summary: efficacy and safety across agents

Efficacy and safety data for these agents derive primarily from phase 2/3 randomized trials and emerging real-world observational cohorts, each with distinct strengths and limitations.

Pradefovir mesylate demonstrated comparable HBV DNA suppression to TDF at Week 24 in a randomized phase 2 noninferiority trial, with acceptable safety profiles in healthy volunteers and treatment-naïve patients PDV demonstrated comparable HBV DNA suppression to TDF in Phase 2 (Week 24) and Phase 3 (Weeks 48 and 96) randomized trials, with acceptable safety profiles and improved bone and renal parameters compared with TDF at Week 96 (Gao et al.). The subsequent phase 3 noninferiority trial (NCT04543565, n = 908) randomized Chinese CHB patients 2:1 to PDV 45 mg or tenofovir disoproxil fumarate 300 mg once daily, and has now been published, providing extended efficacy and safety data (Gao et al., 2026). At Week 48, pradefovir demonstrated noninferior virological suppression versus TDF using a prespecified noninferiority margin of −12%. In HBeAg-positive patients, HBV DNA <29 IU/mL was achieved in 59.5% (264/444) versus 61.3% (136/222) with TDF (difference −1.8%, 95% CI: −9.7 to 6.1); in HBeAg-negative patients, rates were 92.6% (150/162) versus 90.0% (72/80) (difference 2.6%, 95% CI: −5.1–10.3). Among patients with abnormal baseline ALT, normalization occurred in 82.9% (306/369) versus 87.4% (159/182) of HBeAg-positive patients. At Week 96, drug-related adverse events were significantly less frequent with pradefovir than with TDF (58.6% (355/606) versus 71.9% (217/302); difference −13.3%, 95% CI: −19.7 to −6.9; P < 0.0001), with improved bone mineral density preservation and renal safety parameters compared with TDF. However, the genetic barrier to resistance and long-term virologic durability beyond 96 weeks remain incompletely characterized. Safety data in special populations—including those with decompensated cirrhosis, pregnancy, and HBV/HIV coinfection—are insufficient to guide clinical use at present. Consequently, PDV is best positioned as an emerging alternative requiring cautious implementation, rather than as an established therapeutic equivalent to TAF or TMF. Consequently, PDV should be positioned as an additional therapeutic option alongside existing nucleotide prodrugs, with attention to concomitant medications given its CYP3A4-dependent activation.

Tenofovir amibufenamide is supported by more robust evidence, including randomized phase 3 trials demonstrating noninferior antiviral efficacy to TDF at Week 48 TMF is supported by Phase 3 evidence, including randomized trials demonstrating noninferior antiviral efficacy to TDF at Week 48, with sustained improvement in renal and bone safety parameters persisting through Week 96 (Liu et al., 2021; Liu et al., 2023). Prospective real-world studies report largely neutral lipid profiles over 48-week follow-up, with no significant changes in total cholesterol, LDL cholesterol, HDL cholesterol, or triglycerides (Chen et al., 2024; Peng et al., 2023). A 144-week Phase 3 extension study specifically examining the TDF-to-TMF switch found that TDF therapy lowered total cholesterol (4.77–3.97 mmol/L), LDL cholesterol (2.74–2.23 mmol/L), and HDL cholesterol (1.45–1.14 mmol/L) during the first 96 weeks; after switching to TMF, all four lipid fractions gradually normalized and converged to levels comparable to continuous TMF recipients by week 144 (Zeng et al., 2025). When comparing TMF directly with TAF, available data derive from retrospective observational cohorts with limited follow-up duration and inherent potential for selection bias. These real-world analyses suggest comparable antiviral efficacy at 48 weeks. A recent single-center retrospective cohort extended this comparison to patients with HBV-related decompensated cirrhosis, reporting comparable complete virologic response rates (85.7% vs. 90.7%; P = 0.791) and similar improvements in hepatic function between TMF and TAF over 48 weeks (Rong et al., 2025). Regarding lipid profiles, a 48-week retrospective cohort of 215 patients reported that TAF increased total cholesterol by a mean of 35 mg/dL (from 4.30 ± 1.54 mmol/L (166 mg/dL) at baseline to 5.20 ± 0.99 mmol/L (201 mg/dL) at week 48; within-group P = 0.045), whereas TMF remained stable (from 4.83 ± 1.09 mmol/L (187 mg/dL) to 4.82 ± 1.52 mmol/L (186 mg/dL); within-group P = 0.822); the between-group difference in lipid changes did not reach statistical significance (P = 0.182). Regarding lipid profiles, a 48-week retrospective cohort of 215 patients reported divergent within-group changes in total cholesterol: TAF increased by a mean of 35 mg/dL (from 4.30 ± 1.54 mmol/L [166 mg/dL] at baseline to 5.20 ± 0.99 mmol/L [201 mg/dL] at week 48; within-group P = 0.045), while TMF showed minimal change (from 4.83 ± 1.09 mmol/L [187 mg/dL] to 4.82 ± 1.52 mmol/L [186 mg/dL]; within-group P = 0.822). Critically, the direct between-group comparison of lipid changes was not statistically significant (P = 0.182), and no inference of differential metabolic effect or superiority of either agent can be drawn from these within-group findings alone. Additionally, a 24-week propensity score-matched retrospective study in treatment-experienced patients showed a significantly greater total cholesterol increase with TAF than TMF (+16.6 mg/dL vs. −0.8 mg/dL; between-group P = 0.003); in treatment-naive patients, the between-group difference was not significant (+7.7 mg/dL vs. −3.5 mg/dL; P = 0.092). Notably, these comparative lipid findings warrant cautious interpretation given the inherent limitations of retrospective observational design, relatively short follow-up duration, and the potential for unmeasured confounding. Definitive therapeutic equivalence of TMF versus TAF requires prospective randomized head-to-head trials with extended follow-up and standardized lipid assessment protocols. Definitive conclusions regarding differential metabolic effects and lipid safety profiles of TMF versus TAF require prospective randomized head-to-head trials with extended follow-up and standardized lipid assessment protocols.

TAF remains the most extensively validated agent, supported by phase 3 evidence through Week 96 demonstrating noninferior antiviral efficacy versus TDF and improved renal and bone outcomes (Chan et al., 2016; Buti et al., 2016; Agarwal et al., 2018). The broadest body of real-world safety experience exists for TAF in special populations including decompensated cirrhosis and HBV/HIV coinfection. These factors collectively position TAF as the preferred agent in patient populations where PDV and TMF lack adequate safety characterization. These factors currently position TAF as the preferred agent in special populations where PDV and TMF have not yet accumulated equivalent long-term safety data.

### 3.3 Pragmatic clinical selection algorithm

**FIGURE 2:**
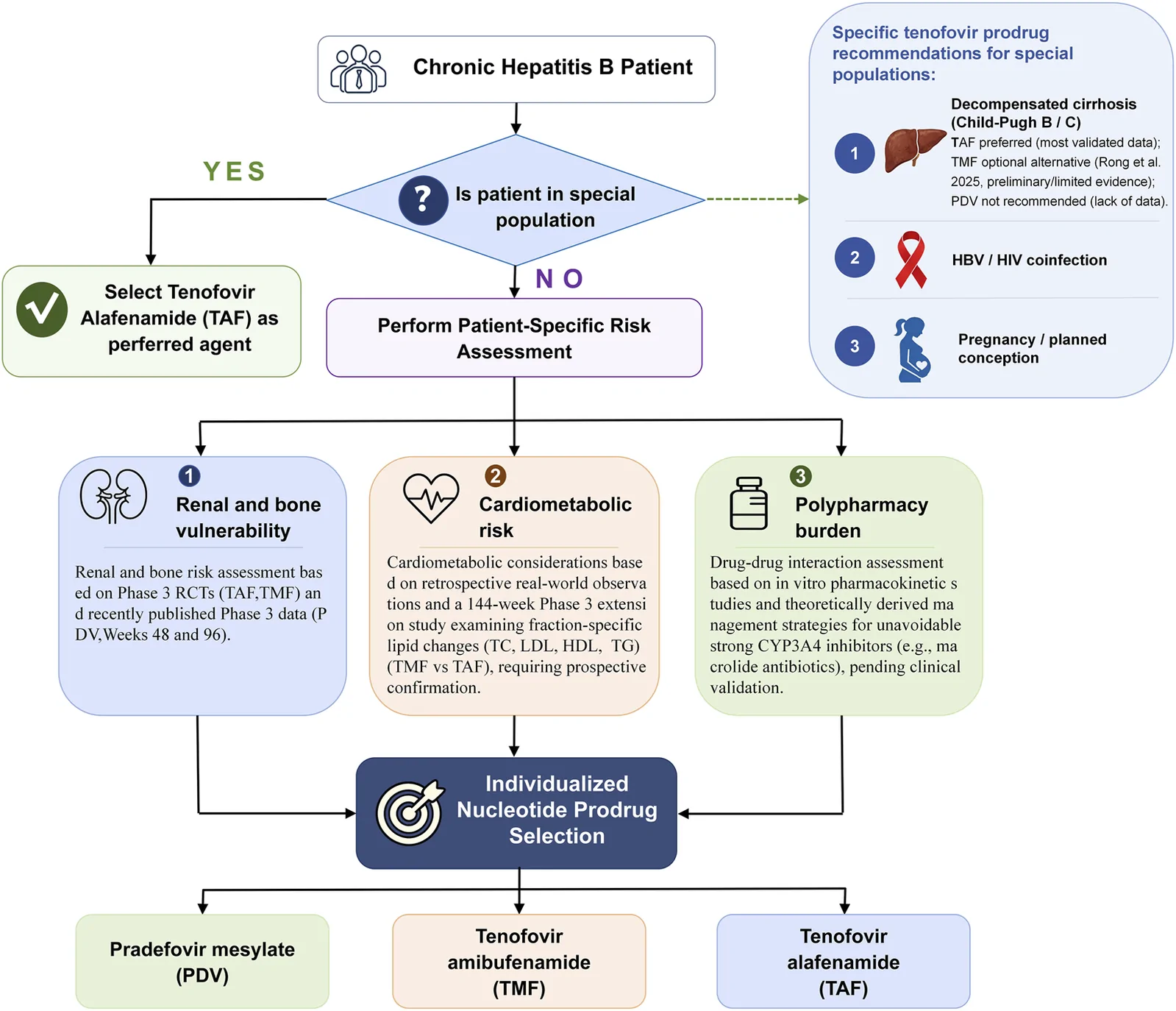
Pragmatic clinical algorithm for selecting pradefovir mesylate, tenofovir amibufenamide, and tenofovir alafenamide in chronic hepatitis B. This bedside flowchart integrates patient-specific risk assessment (renal and bone vulnerability, cardiometabolic risk, polypharmacy burden) to guide individualized nucleotide prodrug selection. Decision nodes are supported by the following evidence: (1) Renal and bone risk assessment based on Phase 3 RCTs (TAF, TMF) and recently published Phase 3 data (PDV, Weeks 48 and 96); (2) Cardiometabolic considerations based on retrospective real-world observations and a 144-week Phase 3 extension study examining fraction-specific lipid changes (TC, LDL, HDL, TG) (TMF vs. TAF), requiring prospective confirmation; (3) Drug-drug interaction assessment based on *in vitro* pharmacokinetic studies and theoretically derived management strategies for unavoidable strong CYP3A4 inhibitors (e.g., macrolide antibiotics), pending clinical validation. Special populations requiring TAF as the preferred agent are highlighted: decompensated cirrhosis (Child-Pugh B/C), HBV/HIV coinfection, and pregnancy/planned conception.

We propose a provisional, pragmatic bedside framework (Figure 2; Table 1) integrating patient-specific risk profiles, antiviral drug characteristics, and pharmacokinetic considerations within the context of current regional and international guidelines (EASL, 2025; Terrault et al., 2018; Chinese Medical Association, 2022). This framework is designed for real-world implementation during the evidence-to-practice transition.

**TABLE 1:** Comparative profiles, clinical positioning, and monitoring framework for nucleotide prodrugs in chronic hepatitis B.

Domain	PDV	TMF	TAF
Active moiety	Adefovir diphosphate (PMEA-DP)	Tenofovir diphosphate	Tenofovir diphosphate
Key clinical evidence	Phase 2 vs. TDF (Week 24); phase 3 vs. TDF (Weeks 48 & 96)	Phase 3 vs. TDF (Weeks 48 & 96)	Phase 3 vs. TDF (Weeks 48 & 96)
Renal/bone safety vs. TDF	Improved bone mineral density preservation and renal safety parameters vs. TDF at Week 96; longer-term data accumulating	Improved renal & bone parameters vs. TDF (phase 3)	Improved renal & bone measures vs. TDF (phase 3)
Lipid profile	Limited data available	Retrospective data: minimal change from baseline; prospective cohorts neutral. Between-group comparison vs. TAF: not statistically significant (P = 0.182). Switching from TDF: TC, LDL, HDL, and TG all normalize by W144 after initial TDF-induced lowering	Retrospective data: TC +35 mg/dL vs. TAF over 48 weeks (P = 0.045); TC +35 mg/dL from baseline over 48 weeks (within-group P = 0.045), vs. TMF stable; +10–20 mg/dL vs. TDF in other reports. Switching from TDF: TC and TG elevations; variable HDL/LDL
Evidence quality: TMF vs. TAF comparison	N/A	Retrospective observational cohorts; requires RCT validation	Established reference
Drug-drug interactions	CYP3A4-dependent activation; screening for strong inhibitors or inducers is recommended based on in vitro data (Ki = 12.5 nM); clinical validation pending	Non-CYP pathway (carboxylesterase 1); low DDI risk	Non-CYP pathway (cathepsin A); low DDI risk
Regulatory status (China)	2024 (approved); 2025 (NRDL inclusion)	2021	2016
Standard dose	45 mg once daily	25 mg once daily	25 mg once daily
Baseline monitoring	HBV DNA, ALT/AST, creatinine/eGFR; CYP3A4 comedication screening recommended	HBV DNA, ALT/AST, creatinine/eGFR; fasting lipids if metabolic risk	HBV DNA, ALT/AST, creatinine/eGFR; fasting lipids (especially if cardiometabolic risk)
Week 12/24 monitoring	HBV DNA + ALT; renal function; reassess DDI status	HBV DNA + ALT; renal labs; lipids if metabolic risk	HBV DNA + ALT; renal function; lipid monitoring
Switch/alert triggers	Unavoidable CYP3A4 DDI risk; confirmed progressive renal decline from baseline	Confirmed progressive renal decline from baseline; new tubular dysfunction; dyslipidemia refractory to lifestyle and pharmacologic management	Confirmed progressive renal decline from baseline; dyslipidemia refractory to lifestyle and pharmacologic management
Special populations*	Insufficient data: decompensated cirrhosis, HBV/HIV coinfection, pregnancy	Insufficient data: decompensated cirrhosis, HBV/HIV coinfection, pregnancy	Preferred: decompensated cirrhosis, HBV/HIV coinfection; extensive pregnancy registry data

For patients receiving PDV who require strong CYP3A4 inhibitors (e.g., macrolides), temporary interruption or switch to TMF or TAF may be considered based on pharmacological theory; dose reduction lacks pharmacologic rationale. Pharmacoeconomic considerations: Generic TAF and TDF represent the most cost-effective options following centralized procurement; TMF and PDV are reimbursed but at substantially higher patient cost-sharing.

Abbreviations: PDV, pradefovir mesylate; TMF, tenofovir amibufenamide; TAF, tenofovir alafenamide; TDF, tenofovir disoproxil fumarate; DDI, drug-drug interaction; eGFR, estimated glomerular filtration rate; RCT, randomized controlled trial.

Before applying this general framework, clinicians should recognize that TAF remains the preferred agent in three specific populations due to insufficient safety data for PDV and TMF: (1) decompensated cirrhosis (Child-Pugh Class B or C); TAF remains the preferred agent in three specific populations: (1) decompensated cirrhosis (Child-Pugh Class B or C), where PDV lacks any safety data and TMF is supported only by limited single-center evidence; (2) HBV/HIV coinfection; and (3) pregnancy or planning for conception. For patients with decompensated cirrhosis (Child-Pugh B/C), neither PDV nor TMF has established dosing strategies or robust safety data in this high-risk population, and pathophysiological considerations further support preferential use of TAF. For patients with decompensated cirrhosis (Child-Pugh B/C), PDV lacks established dosing strategies or safety data. TMF has emerging real-world data (Rong et al., 2025) showing comparable 48-week efficacy (85.7% vs. 90.7% complete virologic response; P = 0.791) and renal safety to TAF, suggesting retained antiviral activity despite theoretical concerns about impaired prodrug activation in advanced liver disease. However, these findings are limited by small sample size (n = 98), single-center design, short follow-up, and lack of pharmacokinetic validation of intrahepatic TFV-DP concentrations or bone mineral density assessment. Pathophysiological considerations therefore support preferential use of TAF, though TMF may be considered as an alternative when TAF is unsuitable. 1n advanced cirrhosis, altered hepatic blood flow, extensive portosystemic shunting, and impaired hepatocyte enzymatic function fundamentally disrupt the targeted intrahepatic activation upon which both prodrugs depend (Doligalski et al., 2012; Bisht et al., 2026). PDV requires obligate CYP3A4-mediated oxidation within hepatocytes; however, in decompensated disease, reduced hepatocellular CYP3A4 activity and portosystemic shunting may decrease hepatic generation of adefovir diphosphate while increasing systemic PMEA exposure, raising renal tubular risk. Similarly, TMF depends on hepatic carboxylesterase 1 (CES1) for activation to tenofovir diphosphate; diminished functional hepatocyte mass and impaired portal perfusion may reduce intrahepatic TFV-DP delivery and increase systemic tenofovir levels, thereby undermining the renal and bone safety advantages engineered into the prodrug. A recent single-center retrospective cohort in HBV-related decompensated cirrhosis reported comparable 48-week complete virologic response rates between TMF and TAF (85.7% vs. 90.7%; P = 0.791), with no significant between-group differences in renal function parameters (Rong et al., 2025). These findings suggest that TMF retains antiviral efficacy and renal safety in this population despite theoretical concerns about impaired prodrug activation. While these findings are encouraging, this study was limited by small sample size (n = 98), single-center design, short follow-up duration, and lack of pharmacokinetic validation of intrahepatic TFV-DP concentrations or bone mineral density assessment. Consequently, TAF remains preferred in this population given its extensive validated safety data despite also relying on hepatocellular enzymatic activation. For HBV/HIV coinfection, TAF (or TDF) is strongly favored due to dual antiviral activity against both HIV and HBV, whereas TMF lacks validated HIV suppression efficacy data and PDV’s CYP3A4 interactions may complicate concurrent antiretroviral regimens. Regarding pregnancy planning, all three agents lack sufficient gestational safety registries; however, TDF has the most extensive pregnancy exposure data and remains the default choice for women considering conception. Pradefovir-specific preclinical reproductive toxicology data have not yet been published; however, as a liver-targeted prodrug of PMEA, its pregnancy risk assessment may be informed by the reproductive toxicology profile of adefovir dipivoxil, which showed no embryotoxicity or teratogenicity in rats and rabbits at 23-fold and 40-fold human exposure, respectively, following oral administration, whereas intravenous adefovir at 38-fold exposure caused embryotoxicity in rats (no adverse effects at 12-fold exposure) (Ltd ECGS, 2016). These data pertain to the active metabolite rather than pradefovir itself, and direct preclinical reproductive studies of pradefovir remain an evidence gap.

Beyond these special populations, renal and bone risk assessment represents a primary determinant of agent selection. Patients at high risk for renal or bone complications—including those with chronic kidney disease, osteoporosis, age older than 65 years with comorbidities, diabetes mellitus or hypertension accompanied by proteinuria, or anticipated treatment duration exceeding ten years—should preferentially receive TMF or TAF based on robust Phase 3 evidence of superior renal and bone safety outcomes based on Phase 3 evidence of improved renal and bone safety profiles compared with TDF (Liu et al., 2021; Liu et al., 2023; Chan et al., 2016; Buti et al., 2016). PDV may be considered only when access to newer agents is severely restricted and when the likelihood of clinically relevant drug-drug interactions is minimal.

When dyslipidemia or elevated atherosclerotic cardiovascular disease risk is a clinical priority—operationally defined as baseline LDL-cholesterol ≥130 mg/dL, metabolic syndrome, or coexisting non-alcoholic fatty liver disease—TMF may be considered based on preliminary real-world observations showing divergent total cholesterol trajectories relative to TAF (e.g., TAF +35 mg/dL over 48 weeks (P = 0.045) vs. TMF stable (P = 0.822); and in treatment-experienced patients, TAF +16.6 mg/dL vs. TMF −0.8 mg/dL at 24 weeks (P = 0.003)). However, these comparative data derive from retrospective observational cohorts with selection bias, baseline lipid imbalances, and limited follow-up; randomized head-to-head trials with standardized lipid protocols are urgently needed to confirm these findings. If TAF is selected in a patient with significant cardiometabolic risk, baseline and early follow-up lipid assessments (e.g., at Week 12) are advisable, with periodic reassessment according to cardiology guidelines Dyslipidemia or elevated atherosclerotic cardiovascular disease risk may be regarded as a clinical priority in patients with baseline LDL-cholesterol ≥130 mg/dL, metabolic syndrome, or coexisting non-alcoholic fatty liver disease. In such patients, clinicians should recognize that available retrospective data report divergent within-group total cholesterol trajectories for TAF and TMF, but direct between-group comparisons have generally failed to demonstrate statistically significant differences (e.g., P = 0.182 at 48 weeks; P = 0.092 in treatment-naive patients at 24 weeks). The single between-group finding of a greater increase with TAF in treatment-experienced patients (+16.6 mg/dL vs. −0.8 mg/dL; P = 0.003) derives from a small, short-term observational study susceptible to selection bias and confounding. Consequently, these data are hypothesis-generating only and do not support preferential selection of either TMF or TAF on metabolic grounds. Regardless of the chosen nucleotide prodrug, baseline and early follow-up lipid assessments (e.g., at Week 12) are advisable for patients with significant cardiometabolic risk, with periodic reassessment according to cardiology guidelines (Peng et al., 2023; Zhang et al., 2015).

Concomitant medication review is particularly important when considering PDV, as strong CYP3A4 inducers or inhibitors may substantially theoretically alter its hepatic bioactivation and generally argue against its use or necessitate warrant specialist consultation. Common CYP3A4 inhibitors include azole antifungals, macrolide antibiotics, protease inhibitors, and selected calcium-channel blockers, while rifampicin and phenytoin are potent inducers. For patients who absolutely require concurrent therapy with strong CYP3A4 inhibitors such as macrolide antibiotics, including clarithromycin or erythromycin, a theoretically grounded management strategy can be proposed, although clinical validation is pending. For patients in whom concurrent therapy with strong CYP3A4 inhibitors (e.g., macrolide antibiotics) is unavoidable, a management strategy grounded in pharmacological theory may be considered, although clinical validation is pending. Because macrolides potently inhibit CYP3A4 mediated conversion of PDV to its active moiety, with a Ki of 12.5 nM, co-administration risks subtherapeutic intrahepatic drug delivery and potential virologic breakthrough. Because macrolides potently inhibit CYP3A4-mediated conversion of PDV to its active moiety *in vitro* (Ki = 12.5 nM), co-administration could theoretically result in subtherapeutic intrahepatic drug delivery and possible virologic breakthrough. In this scenario, temporary interruption of PDV for the duration of antibiotic therapy (typically 5–14 days) may be considered a pragmatic theoretical option, typically 5–14 days, is the preferred theoretical approach, given that the risk of HBV reactivation during such a brief treatment holiday is low in compensated, virologically suppressed patients, whereas dose reduction lacks pharmacologic rationale because it would further diminish active metabolite generation. If treatment interruption is deemed unsafe, for example, in patients with recent virologic relapse or documented resistance history, switching to TMF or TAF, both of which utilize non-CYP activation pathways, for the antibiotic treatment period represents a mechanistically sound alternative. Upon macrolide discontinuation, PDV may be resumed at the standard 25 mg 45 mg dose without re-titration. These recommendations These considerations are derived from *in vitro* metabolic data and principles of CYP3A4 pharmacology; prospective DDI studies in CHB cohorts are urgently needed to confirm their clinical validity. In contrast, TMF and TAF have demonstrated no clinically significant drug-drug interaction concerns in CHB monotherapy and offer substantially greater pharmacologic flexibility in polypharmacy scenarios.

We explicitly acknowledge that pharmacoeconomic realities substantially influence the real-world feasibility of any clinical selection algorithm in China. As of 2025–2026, TMF is listed in the National Reimbursement Drug List (NRDL) at a negotiated price of approximately 456 RMB per box (25 mg × 30 tablets), with patient out-of-pocket costs of ∼10% as a Class B reimbursed drug. PDV, approved in October 2024 and included in the 2025 NRDL update (effective January 2026), is positioned at a comparable price point to TMF as a Class B reimbursed agent. In contrast, TAF presents a bifurcated economic landscape: while the originator remains at ∼540 RMB/month, domestic generics have entered centralized procurement at dramatically reduced prices—as low as 9.6 RMB per box (25 mg × 30 tablets) in the 7th National Volume-Based Procurement, yielding a monthly cost of <10 RMB. Consequently, for treatment-naïve patients without specific renal, bone, metabolic, or drug-interaction risk factors, generic TAF or TDF (annual cost ∼100–200 RMB) represents the most economically accessible choice treatment-naïve patients without specific risk factors, TDF or generic TAF represent effective, guideline-endorsed, and economically accessible first-line options. Our algorithm is intended to guide individualized selection when safety or pharmacokinetic profiles favor newer agents (e.g., chronic kidney disease, osteoporosis, anticipated >10-year therapy, or mandatory CYP3A4 comedication screening for PDV), not to mandate costlier therapy when clinical equivalence exists at lower cost. Local formulary constraints, insurance reimbursement tiers, and patient cost-sharing must inform final prescribing decisions.

Implementation of antiviral therapy should follow standardized baseline laboratory assessment (Table 1). Virologic and safety monitoring should occur at Week 12 and Week 24 to establish baseline response and safety tolerability. In virologically suppressed, clinically stable patients, subsequent monitoring at 24–48-week intervals is generally sufficient. Switching among nucleotide prodrugs may be considered in the setting of (1) a confirmed, progressive decline in renal function from baseline, particularly if accompanied by new-onset proteinuria, proximal tubular dysfunction, or a fall into a lower KDIGO chronic kidney disease stage; (2) emergence of proximal tubular dysfunction; (3) persistent or clinically relevant dyslipidemia despite optimal cardiovascular management; (4) unavoidable exposure to CYP3A4-modifying agents (particularly relevant for PDV); or (5) patient preference and cost considerations where therapeutic interchange is permitted by regulatory bodies or payers. Figure 2 provides a visual implementation flowchart synthesizing these decision points.

### 3.4 Study limitations

The certainty of evidence underlying key conclusions was formally assessed using GRADE, with detailed ratings provided in Supplementary Material Table S1. Randomized Phase 3 trials of TMF and TAF versus TDF provided moderate-certainty evidence for antiviral efficacy and renal/bone safety, downgraded for indirectness and industry sponsorship. Randomized Phase 3 trials comparing TMF and TAF with TDF provided moderate-certainty evidence for the efficacy and renal/bone safety of these newer agents. TDF itself remains the most extensively validated comparator with the longest real-world safety record and the highest barrier to resistance. This indirectness reflects the current absence of direct head-to-head trials comparing all three agents simultaneously. Pradefovir efficacy data were rated as low-to-moderate certainty given Phase 2 origins and recently published but shorter-term Phase 3 follow-up. Real-world TMF-versus-TAF comparisons were uniformly rated as low or very low certainty due to retrospective design, selection bias, short follow-up, and lack of standardized lipid protocols. Beyond these evidence-quality constraints, this narrative review did not perform a systematic literature search with formal risk-of-bias assessment or quantitative meta-analysis, which constrains the strength of comparative inferences. Beyond these evidence-quality constraints, quantitative meta-analysis was not performed due to significant clinical heterogeneity across study designs and the absence of direct head-to-head trials comparing all three agents simultaneously, which constrains the strength of pooled comparative inferences. Moreover, our cross-agent comparisons rely on indirect inferences anchored to TDF as a common comparator, precluding definitive claims of therapeutic equivalence or superiority. Moreover, cross-agent comparisons rely on indirect inferences anchored to TDF as a common comparator, precluding definitive claims of therapeutic equivalence or superiority. Finally, the available Phase 3 evidence derives predominantly from Chinese cohorts, limiting generalizability to non-Asian populations, different HBV genotypes, and healthcare systems with divergent comorbidity profiles.

### 3.5 Evidence gaps and future directions

While the proposed algorithm synthesizes current best evidence, substantial uncertainties warrant explicit acknowledgment and will continue to shape clinical practice as evidence matures.

The comparative data between TMF and TAF derive largely from retrospective real-world cohorts conducted over limited follow-up periods of 48 weeks, introducing inherent limitations in study design. These analyses suggest comparable antiviral efficacy and potentially differential lipid effects; however, such findings are vulnerable to selection bias, unmeasured confounding, and variability in lipid measurement protocols across centers and time points (Peng et al., 2023; Li et al., 2023). To definitively establish whether the apparent lipid advantages of TMF apparent divergence in lipid trajectories between TMF and TAF represent genuine pharmacologic differences or merely artifacts of patient selection and monitoring practices, randomized head-to-head Phase 3 trials are essential. Such trials should specifically examine cardiometabolic safety trajectories, long-term renal and bone outcomes beyond 96 weeks, and stratified analyses by HBeAg status, baseline fibrosis stage, and metabolic comorbidities. Only with such rigorous prospective data can clinicians confidently transition TMF from an “emerging alternative” to a true therapeutic equivalent of TAF.

For PDV, while a recent Phase 3 trial comparing PDV to TDF in Chinese CHB patients has been published, extending the evidence base beyond the initial Week 24 data, critical knowledge gaps remain. The genetic barrier to HBV resistance during PDV therapy has not been systematically characterized, raising questions about long-term virologic durability and the potential for treatment failure due to resistance emergence. Clinical validation of the predicted CYP3A4-mediated drug-drug interactions remains pending in real-world CHB populations receiving polypharmacy; without such validation, prescribers must rely on theoretical predictions rather than empirical safety data when encountering patients on concurrent CYP3A4-modifying medications. Safety and efficacy data in special populations—those with decompensated cirrhosis, HBV/HIV coinfection, and pregnancy—are entirely insufficient, preventing evidence-based dose recommendations or monitoring strategies in these high-risk groups. Head-to-head trials comparing PDV directly with TMF and TAF would further elucidate whether its unique CYP3A4-dependent bioactivation and favorable hepatic selectivity confer clinical advantages that justify the added complexity of mandatory drug-drug interaction screening.

Pharmacoeconomic validation remains a critical unmet need. The feasibility of any selection algorithm depends on cost-effectiveness evidence within China’s healthcare system. Generic TAF and TDF now cost <10 RMB/month and ∼100–200 RMB/year, respectively, following centralized procurement, whereas reimbursed TMF and PDV remain at ∼456 RMB/month—a disparity that profoundly shapes real-world prescribing. Comparative analyses should integrate direct drug costs, monitoring expenditures, and long-term complication expenses (renal replacement therapy, fracture management, cardiovascular events) to determine whether incremental safety benefits justify higher acquisition costs in specific risk strata. Implementation research examining prescriber adherence when algorithmic recommendations conflict with formulary constraints is equally necessary. Without such economic and operational validation, clinical frameworks risk remaining theoretical rather than operational.

Beyond individual agent-specific gaps, several broader research priorities merit emphasis. Prospective studies are urgently needed in patient populations systematically underrepresented in current trials, including those with advanced cirrhosis (Child-Pugh B/C), HBV/HIV coinfection, advanced renal disease (eGFR <30 mL/min/1.73 m^2^), and pregnancy. Pharmacoeconomic analyses from diverse Asia-Pacific healthcare systems would clarify the cost-effectiveness and real-world affordability of different therapeutic sequencing and switching strategies, incorporating direct medical costs and indirect societal costs. Mechanistic investigations examining HBV DNA kinetics during early therapy, the emergence and evolution of resistance-associated variants over extended follow-up, and long-term immunologic outcomes (T-cell exhaustion, regulatory T-cell dynamics) during therapy with TMF, PDV, and TAF would provide deeper insights into durability and may identify biomarkers predictive of clinical response and safety. Standardized lipid measurement protocols and consistent reporting of cardiometabolic outcomes across future trials would enable valid meta-analyses and stronger evidence synthesis. Similarly, the standardization of renal monitoring protocols and switching thresholds among nucleotide prodrugs remains to be established. Current Phase 3 trials have demonstrated differential renal safety profiles but have not harmonized the eGFR thresholds or confirmatory testing requirements that should trigger therapeutic switching (Mak et al., 2026). Once these research priorities are systematically addressed, the clinical algorithm presented herein will be refined and updated to reflect stronger evidence and more precisely defined therapeutic positioning (Grundy et al., 2018; Stone et al., 2013).

## 4 Conclusion

Among novel hepatocyte-targeted nucleotide prodrugs, TMF is supported by robust phase 3 evidence extending to 96 weeks demonstrating noninferior antiviral efficacy to TDF and superior improved renal and bone safety profiles. Retrospective real-world comparisons with TAF demonstrate comparable antiviral efficacy; however, definitive therapeutic equivalence requires prospective randomized trials with extended follow-up and standardized monitoring protocols. Pradefovir mesylate represents a promising therapeutic option with comparable antiviral activity to TDF at Week 24 and now supported by phase 3 data PDV offers comparable antiviral activity to TDF through Week 96 (Gao et al., 2026); however, its long-term durability, genetic barrier, and drug-drug interaction profile require fuller characterization before integration into standard treatment algorithms. with favorable renal and bone safety findings at that timepoint. Its CYP3A4-dependent activation warrants medication review at initiation, and continued long-term follow-up will further define its positioning among nucleotide prodrugs. TAF remains the guideline-endorsed benchmark with the most extensive safety experience across special populations (Chan et al., 2016; Buti et al., 2016; Agarwal et al., 2018). The pragmatic bedside algorithm presented herein integrates renal and bone risk, metabolic status, and drug-drug interaction potential to guide individualized nucleotide prodrug selection (EASL, 2025; Terrault et al., 2018; Chinese Medical Association, 2022), but should be updated as additional Phase 3 trials, head-to-head comparative studies, and pharmacoeconomic analyses emerge from the Asia-Pacific region and beyond. Pending such evidence, the recommendations herein remain provisional. Finally, it is important to emphasize that TDF remains the global standard of care with the highest barrier to resistance, the most extensive real-world safety experience, and the lowest cost. The newer hepatocyte-targeted prodrugs discussed herein provide incremental therapeutic options for specific risk populations but do not replace TDF as the foundational first-line therapy for chronic hepatitis B.

## Data Availability

The data extracted and analyzed in this systematic review are included in the article and its Supplementary Material. Further inquiries can be directed to the corresponding author.
