# Supplementary material for: Pradefovir mesylate and tenofovir amibufenamide for chronic hepatitis B: a systematic review and pragmatic treatment algorithm
Source: Front Pharmacol. 2026 Aug 24;17:1862886. doi: 10.3389/fphar.2026.1862886 (PMC13547824; doi:10.3389/fphar.2026.1862886)
Supplement: Supplementary file 1 [file Table1.docx]

**Supplementary Table 1. GRADE certainty of evidence assessments for major clinical conclusions**

| Clinical Conclusion | Evidence Base (References) | Study Design | Overall GRADE Rating | Domains for Downgrading | Explanation |
| --- | --- | --- | --- | --- | --- |
| **TMF vs TDF: Antiviral efficacy** (Weeks 48 & 96) | Liu et al. 2021; Liu et al. 2023 | Phase 3 RCT | Moderate | Indirectness; Industry sponsorship | No head-to-head comparison with TAF; evidence anchored to TDF as common comparator; industry-sponsored trials |
| **TMF vs TDF: Renal and bone safety** (Weeks 48 & 96) | Liu et al. 2021; Liu et al. 2023 | Phase 3 RCT | Moderate | Indirectness; Industry sponsorship | Superior to TDF established; no direct TMF-vs-TAF comparison; industry-sponsored trials |
| **TAF vs TDF: Antiviral efficacy** (Weeks 48 & 96) | Chan et al. 2016; Buti et al. 2016; Agarwal et al. 2018 | Phase 3 RCT | Moderate | Indirectness; Industry sponsorship | No head-to-head comparison with TMF; evidence anchored to TDF comparator; industry-sponsored trials |
| **TAF vs TDF: Renal and bone safety** (Weeks 48 & 96) | Chan et al. 2016; Buti et al. 2016; Agarwal et al. 2018 | Phase 3 RCT | Moderate | Indirectness; Industry sponsorship | Superior to TDF established; no direct TMF-vs-TAF comparison; industry-sponsored trials |
| **PDV vs TDF: Antiviral efficacy** (Weeks 48 & 96) | Gao et al. 2022; Gao et al. 2026 | Phase 2/3 RCT | Low to moderate | Imprecision; Indirectness | Phase 2 origins; recently published but shorter-term Phase 3 follow-up; Chinese population only |
| **TMF vs TAF: Comparative efficacy and safety** (including lipid profiles) | Peng et al. 2023; Li et al. 2023; Rong et al. 2025; Zeng et al. 2025 | Retrospective cohorts | Low or very low | Risk of bias; Indirectness | Retrospective design; selection bias; short follow-up (24–48 weeks); lack of standardized lipid protocols |

Note. GRADE ratings reflect the certainty of evidence for each major conclusion as explicitly stated in Section 3.4 of the main text. RCT, randomized controlled trial.

**Supplementary Table 2. Quality assessment of included studies**

| Study  (Reference No.) | Study Design | Assessment Tool | Quality Rating | Key Limitations |
| --- | --- | --- | --- | --- |
| Gao et al. 2022 | Phase 2 RCT; 24 weeks† | Cochrane RoB 2 | Low risk | Phase 2 design; short follow-up |
| Gao et al. 2026 | Phase 3 RCT | Cochrane RoB 2 | Low risk | Chinese population only |
| Liu et al. 2021 | Phase 3 RCT | Cochrane RoB 2 | Low risk | 48-week primary endpoint; TDF comparator |
| Liu et al. 2023 | Phase 3 RCT | Cochrane RoB 2 | Low risk | 96-week follow-up |
| Chan et al. 2016 | Phase 3 RCT | Cochrane RoB 2 | Low risk | HBeAg-positive population; TDF comparator |
| Buti et al. 2016 | Phase 3 RCT | Cochrane RoB 2 | Low risk | HBeAg-negative population; TDF comparator |
| Agarwal et al. 2018 | Phase 3 RCT | Cochrane RoB 2 | Low risk | 96-week follow-up; TDF comparator |
| Chen et al. 2024 | Prospective cohort | NOS | High quality (8/9) | Single-center; small sample size (n=91); 48-week follow-up; lipid-focused endpoints only; no in vivo validation |
| Peng et al. 2023 | Retrospective cohort | NOS | High quality (8/9) | Single-center; small sample size (n=215); 48-week follow-up; no bone safety data |
| Li et al. 2023 | Retrospective cohort; 24 weeks† | NOS | High quality (7/9) | Single-center; small sample size; short follow-up; no renal tubular/bone data |
| Lin et al. 2024 | Retrospective cohort | NOS | High quality (8/9) | Single-center; lack of smoking/alcohol/BMI data; no hepatic steatosis verification |
| Rong et al. 2025 | Retrospective cohort | NOS | High quality (7/9) | Single-center; retrospective design; small sample size (n=98); 48-week follow-up; limited to decompensated cirrhosis population |
| Zeng et al. 2025 | Retrospective cohort; Switch-study | NOS | Moderate quality (6/9) | Small sample size (n=53); single-center; high loss to follow-up; post-hoc analysis |

Note: NOS, Newcastle-Ottawa Scale; RoB 2, Cochrane Risk of Bias 2.0; RCT, randomized controlled trial. Quality ratings for observational studies represent conservative estimates based on study design and population characteristics reported in the published titles/abstracts.

† Stratified follow-up criteria: Phase 2 RCTs were eligible with ≥24-week follow-up. Head-to-head retrospective cohorts directly comparing TMF versus TAF were eligible with ≥24-week follow-up given the absence of randomized head-to-head evidence. All other Phase 3 trials, prospective cohorts, and retrospective studies required ≥48-week follow-up.

**Supplementary Table 3. Electronic database search strategies and yield**

| Database (Provider) | Search strategy | Limits / Filters | Records, *n* |
| --- | --- | --- | --- |
| **PubMed** (NCBI) | ((("pradefovir"[tiab] OR "pradefovir mesylate"[tiab] OR "tenofovir amibufenamide"[tiab] OR "tenofovir alafenamide"[tiab] OR "tenofovir disoproxil fumarate"[tiab]) AND ("Hepatitis B, Chronic"[mh] OR "Hepatitis B virus"[mh] OR "chronic hepatitis b"[tiab] OR "hepatitis b virus"[tiab])) NOT ("Vaccines"[mh] OR "Vaccination"[mh] OR "Diagnostic Techniques and Procedures"[mh] OR "Diagnostic Imaging"[mh] OR "Animal Experimentation"[mh] OR "Disease Models, Animal"[mh] OR "in vitro"[tiab] OR "Cells, Cultured"[mh])) AND 2000/01/01:2025/12/31[dp] AND english[la] AND humans[mh] | English; Humans; 2000–2025 | 658 |
| **Embase** (Elsevier) | ('pradefovir':ti,ab,kw OR 'pradefovir mesylate':ti,ab,kw OR 'tenofovir amibufenamide':ti,ab,kw OR 'tenofovir alafenamide':ti,ab,kw OR 'tenofovir disoproxil fumarate':ti,ab,kw) AND ('chronic hepatitis b'/exp OR 'hepatitis b virus'/exp OR 'chronic hepatitis b':ti,ab,kw OR 'hepatitis b virus':ti,ab,kw) NOT ('vaccine'/exp OR 'vaccination'/exp OR 'diagnostic procedure'/exp OR 'diagnostic imaging'/exp OR 'animal experiment'/exp OR 'animal model'/exp OR 'in vitro study'/exp OR 'cell culture'/exp) AND [2000-2025]/py AND english:la | English; 2000–2025 | 632 |
| **Web of Science** (Clarivate) | TS=("pradefovir" OR "pradefovir mesylate" OR "tenofovir amibufenamide" OR "tenofovir alafenamide" OR "tenofovir disoproxil fumarate") AND TS=("Hepatitis B, Chronic" OR "chronic hepatitis B" OR "Hepatitis B virus") AND WC=(Gastroenterology & Hepatology OR Infectious Diseases OR Pharmacology & Pharmacy OR Medicine) NOT TS=(vaccine OR vaccination OR "in vitro") AND PY=(2000-2025) AND LA=(English) AND DT=(Article OR Review) | English; 2000–2025; Article/Review; Subject categories | 1198 |
| **Cochrane Library** (Wiley) | Step 1: ("pradefovir" OR "pradefovir mesylate" OR "tenofovir amibufenamide" OR "tenofovir alafenamide" OR "tenofovir disoproxil fumarate"):ti,ab,kw Step 2: ("Hepatitis B, Chronic" OR "chronic hepatitis B" OR "Hepatitis B virus"):ti,ab,kw Step 3: [mh "Humans"]< Step 4: #1 AND #2 AND #3 | Humans | 170 |
| **CNKI** (Tsinghua Tongfang) | (SU%=pradefovir OR SU%=pradefovir mesylate OR SU%=甲磺酸普雷福韦 OR SU%=替诺福韦艾拉酚胺 OR SU%=替诺福韦酯 OR SU%=艾米替诺福韦 OR SU%=替诺福韦阿米酚胺 OR TI%=pradefovir OR TI%=pradefovir mesylate OR TI%=甲磺酸普雷福韦 OR TI%=替诺福韦艾拉酚胺 OR TI%=替诺福韦酯 OR TI%=艾米替诺福韦 OR TI%=替诺福韦阿米酚胺 OR KY=pradefovir OR KY=pradefovir mesylate OR KY=甲磺酸普雷福韦 OR KY=替诺福韦艾拉酚胺 OR KY=替诺福韦酯 OR KY=艾米替诺福韦 OR KY=替诺福韦阿米酚胺) AND (SU%=慢性乙型肝炎 OR SU%=慢性乙肝 OR SU%=乙型肝炎病毒 OR SU%=HBV OR TI%=慢性乙型肝炎 OR TI%=慢性乙肝 OR TI%=乙型肝炎病毒 OR TI%=HBV OR KY=慢性乙型肝炎 OR KY=慢性乙肝 OR KY=乙型肝炎病毒 OR KY=HBV) AND YE BETWEEN('2000','2025') | 2000–2025 | 705 |
| **VIP** (Chongqing VIP) | (T=pradefovir OR T=pradefovir mesylate OR T=甲磺酸普雷福韦 OR T=替诺福韦艾拉酚胺 OR T=替诺福韦酯 OR T=艾米替诺福韦 OR T=替诺福韦阿米酚胺) AND (T=慢性乙型肝炎 OR T=慢性乙肝 OR T=乙型肝炎病毒) | — | 1249 |

Note.

Abbreviations: tiab, title and abstract; mh, Medical Subject Headings (MeSH); exp, exploded Emtree term; ti, title; ab, abstract; kw, keyword; dp, date of publication; la, language; py, publication year; SU, subject; TI, title; KY, keyword; TS, topic (title, abstract, author keywords, and Keywords Plus); WC, Web of Science category; DT, document type.

Databases were searched on [insert date, e.g., Feb 25, 2026].

Search strategies were tailored to each database’s specific syntax and controlled vocabulary. PubMed used MeSH terms with automatic explosion; Embase used Emtree terms with /exp; Web of Science, CNKI, and VIP used free-text terms.

PubMed and Embase explicitly excluded vaccine, diagnostic, and animal studies via NOT operators. Web of Science restricted results to clinical/pharmacological subject categories (WC) and document types (Article or Review). Chinese databases (CNKI and VIP) do not support human/animal or language filtering in their advanced search interfaces.

The Cochrane Library strategy was constructed stepwise in the Search Manager (Steps 1–3) and combined in Step 4. No publication date limit was applied to the Cochrane query.

The VIP strategy was restricted to the title field (T=) to improve precision and did not include a publication year limit.

Total records retrieved across all six databases before deduplication: 4,612.
